# Correlation between social media addiction and academic procrastination in medical students at public and private medical colleges at Peshawar

**DOI:** 10.12669/pjms.41.3.9276

**Published:** 2025-03

**Authors:** Khurram Naushad, Brekhna Jamil, Naveed Afzal Khan, Maidha Jadoon

**Affiliations:** 1 Dr. Khurram Naushad, MBBS, MHPE Lecturer, Department of Medical Education, Khyber Girls Medical College, Peshawar, Pakistan; 2 Dr. Brekhna Jamil, BDS, MHPE, PhD Medical Education Professor (Director), Institute of Health Professions Education & Research, Khyber Medical University, Peshawar, Pakistan; 3 Dr. Naveed Afzal Khan, MBBS, MHPE, Assistant Professor, Department of Medical Education, Khyber Girls Medical College, Peshawar, Pakistan; 4 Dr. Maidha Jadoon, BDS, MHPE. Assistant Professor, DMER, Women Medical and Dental College, Abbottabad, Pakistan

**Keywords:** Academic procrastination, Medical education, Medical students, Social media addiction

## Abstract

**Objective:**

The study aim was to assess correlation between social media addiction and academic procrastination among medical students at public and private medical colleges in Peshawar.

**Methods:**

A correlational research design was employed with research protocols approved by the Advance Studies and Research Board of Khyber Medical University. Data were collected from two public and five private medical colleges. Universal sampling was then performed. Of 4716 medical students, 3366 participated in the study, with a response rate of 71.4%. This study was conducted between June and December 2023. Data were collected using the structured social media addiction scale (SMAS) and academic procrastination scale (APS).

**Results:**

Male participants were (1775) 52.7%, and the rest (1591) 47.3% were female. Among the responders (1591) 61% resided in hostels. The mean academic achievement was 74.14%, ranging from 30% to 85%. Most medical students (63%) showed moderate and 34% showed high academic procrastination. Majority of the medical students (81%) were social media addicts and male reported higher scores. Social media addiction was highly correlated with academic procrastination (r=0.539), which was regressed and found that 29% (R^2^=0.289) of social media addiction contributed to academic procrastination. Medical students from the public sector, Android users, third-year students, male students, and hostel residents were more prone to social media addiction and academic procrastination.

**Conclusion:**

Social media addiction prevails among medical students and is highly correlated with academic procrastination. Hostel residents, males, and android users were more addicted to social media than their counterparts.

## INTRODUCTION

Social Media (SM) is becoming an important element in our daily lives, with millions of users actively engaging with various platforms, such as Facebook, Twitter, Instagram, and TikTok. The advent of social media has yielded numerous advantages, such as expediting communication, augmenting connectivity, and enabling quick dissemination of knowledge. However, SM has also been found to have adverse effects, including contribution towards procrastination, a common problem experienced by many individuals, including medical students.^1^

Social media addiction (SMA) refers to individuals becoming disproportionately dependent on social media platforms to the point where they affect their daily activities and interfere with their ability to function in various aspects of their lives.^2^ Addiction manifests as spending excessive time on SM, experiencing intense feelings of anxiety or distress when unable to access SM, or neglecting responsibilities, such as work or personal relationships.^3^ Social media addiction can have negative consequences for mental health, including anxiety, despair, and low self-esteem. It can also lead to physical health problems, such as poor sleep, eye strain, and neck and back pain.^4^

Medical students are often overwhelmed by numerous academic tasks, including studying, attending classes, participating in clinical rotations, and engaging in research projects.^5^ The high demand for medical education can create significant stress, leading to procrastination as a coping mechanism. In this context, social media presents an attractive distraction for medical students with its ability to provide instant gratification and entertainment. SM use among medical students is triggered by a variety of factors, including personality traits, time management skills, and academic demands.^6^

Moreover, academic demands, such as a high workload and intense pressure, can contribute to social media use as a form of escape from the stress of medical education. For instance, after many hours of studying, medical students may feel exhausted and seek temporary relief on social media platforms. Although social media can provide temporary relief, its excessive use can lead to procrastination, which adversely influences academic scores. Studies have identified that excessive SM use is highly related to lower academic achievement, decreased motivation, and poor time management skills among medical students.^7^,^8^

Procrastination is the act of delaying or postponing a task or action despite knowing that it will likely result in negative consequences. It is a common behavior that can affect people in many areas of their lives, from work and school to personal relationships and health. There are several explanations regarding procrastination among students, fear of failure, and difficulty concentrating, which lead to a lack of motivation. Procrastination among students is a leading cause of increased stress, anxiety, and guilt as well as a sense of loss of control and productivity.^9^ This study aimed to assess correlation between social media addiction and academic procrastination in the medical students at public and private medical colleges in Peshawar.

## METHODS

This study based on correlational study design was conducted at two public and five private medical colleges in Peshawar between June and December 2023. Medical students at two public and five private medical colleges. Universal Sampling was employed to invite all medical students (n=4716) who were studying at the time of data collection in seven medical colleges to participate in the study. The study was approved by the Institute of Health Professions Education and Research (Ref No.: 1-11/IHPER/MHPE/23-47), dated June 20, 2023.

The data were collected through SMAS (Social Media Addiction Scale (SMAS) and Academic Procrastination Scale (APS), along with demographic information. The SMAS comprises of 29-items. Each item was rated on a five-point Likert scale. The score ranges from 29 to 145; the scores are standardized on a scale of 0-100, with a score of > 50% as a social media addict.^10^ APS comprises of 21-items answered on a five points Likert scale. The score on the scale ranges from 21 to 105; a higher score indicates that medical students perceive themselves as procrastinators. The scale was categorized based on the mean score as ≤ 2.38 low, 2.39 – 3.57 moderate, and ≥ 3.58 high.^11^ Medical students were briefed on the participant information sheet. Informed consent was obtained along with the data collection.

## RESULTS

Of 4716 medical students, 3366 participated in the study, with a response rate of 71.4%. The gender composition of the respondents showed 52.7% as male and 47.3% as female. A large proportion (61.0%) were residents of hostels. Public Sector medical students were (1588) 47.2% of the participants. The respondents showed a fairly even distribution, with approximately 20% each academic year. The demographic characteristics of the participants are presented in Table-I.

**Table-I:** Demographic information of medical students (n=3366).

Demographic Variables		Frequency	Percent
Sex	Male	1775	52.7
	Female	1591	47.3
Residence	Home	1312	39.0
	Hostel	2054	61.0
Medical Colleges	Public – A	458	13.6
	Public – B	1130	33.6
	Private – A	321	9.5
	Private – B	226	6.7
	Private – C	290	8.6
	Private – D	485	14.4
	Private – E	456	13.5
Study Year	1st	730	21.7
	2^nd^	672	20.0
	3^rd^	710	21.1
	4^th^	637	18.9
	5^th^	617	18.3

Students spent an average of 5.76 hours per day on social media, and the average monthly expenditure on internet services was Rs.1460.80. Lastly, the mean score on the previous exam was 72.14%, ranging from 30% to 85%.

**Fig.1:**
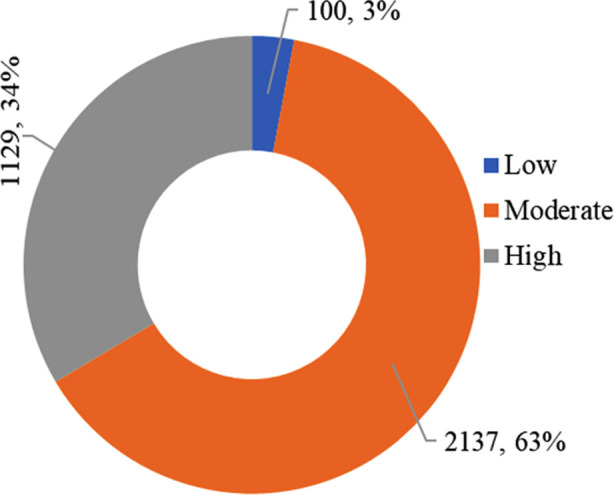
Levels of academic procrastination among medical students (n=3366).

The majority of the students (63%) exhibited moderate levels of academic procrastination, and 34% had high levels, indicating that they tend to frequently delay their work, which can have consequences for their productivity and academic performance, Fig.1.

**Fig 2:**
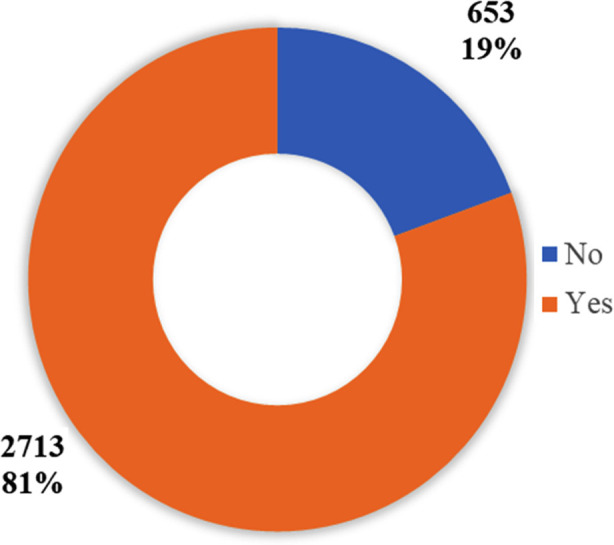
Prevalence of SMA among medical students (n=3366).

In Fig.2 sheds light on SMA levels among a group of medical students. Notably, a substantial majority, representing 81% of charted medical students, falls into the category of social media advertising. This suggests that a significant portion of this medical student cohort exhibits behaviors indicative of social media addiction. The results indicated a statistically significant difference in social media addiction between males and females (p < 0.001), with males showing a higher mean score. However, there was no significant difference in academic procrastination between sexes (p = 0.426), Table-II.

**Table II:** Comparison of Social media addiction and academic procrastination among gender

Scales	Male (n=1775)	Female (n=1591)	t-statistic	Df	P-Value
	Mean±SD	Mean±SD			
Social media addition	3.14±0.66	2.99±0.80	5.65	3364	<0.001
Academic Procrastination	3.28±0.60	3.27±0.56	0.79	3364	0.426

There was a negative correlation between age and the number of hours spent on social media per day (R-value = -0.122), indicating that, on average, as individuals grew older, they tended to spend less time on social media. Regarding academic performance, there was a negative correlation between marks and age (R-value = -0.151), implying that, on average, as students aged, their grades tended to decrease, although this correlation was weak. Furthermore, there was a negative correlation between social media addiction and age (R-value = -0.092), indicating that older individuals are less prone to social media addiction. Notably, a strong and positive correlation existed between social media addiction and the number of hours spent on social media per day (R-value = 0.410). This indicates that individuals who spend more time on social media are more likely to exhibit signs of social media addiction. A substantial positive correlation was also observed between social media addiction and the amount of money spent on Internet services per month (R-value = 0.323), suggesting that those who spend more on Internet services may be more prone to social media addiction. Moreover, there was a statistically significant correlation between social media addiction and the scores (R-value = 0.131), indicating that individuals with higher levels of social media addiction tended to have lower scores, Table-III.

**Table-III:** Correlation matrix (n=3366).

	Pearson’s Value	Age	Hrs/Day on social media	PKR/Month for internet	Marks	Social Media Addiction
Hrs/Day on social media	R-value	-0.122*				
	P-Value	0.001				
PKR/Month for internet	R-value	-0.115*	0.362*			
	P-Value	0.001	0.001			
Marks	R-value	-0.151*	0.013	0.120*		
	P-Value	0.001	0.065	0.001		
Social Media Addiction	R-value	-0.092*	0.410*	0.323*	0.131*	
	P-Value	0.001	0.001	0.001	0.001	
Academic Procrastination	R-value	-0.040*	0.166*	0.231*	0.115*	0.539*
	P-Value	0.021	0.001	0.001	0.001	0.001

* Correlation is significant at the 0.05 level (2-tailed).

**Fig 3:**
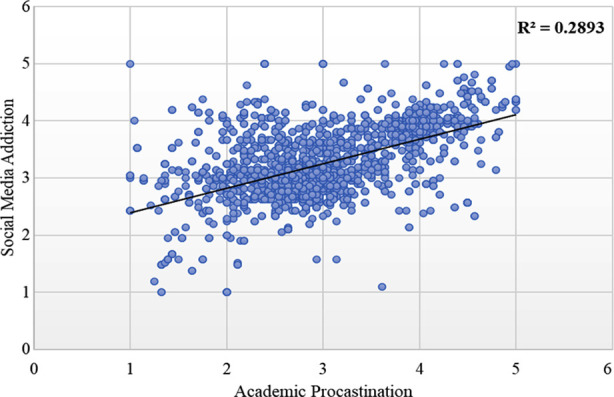
Linear Regression between social media addiction and procrastination

The linear regression result indicated that there was a linear relationship between social media addiction and procrastination, and the coefficient of determination (R-squared value) was 0.2893. Approximately 28.93% of the variation in procrastination can be attributed to variations in social media addiction. This finding suggests a moderately strong relationship between social media addiction and procrastination. The remaining 71.07% of the variance in procrastination was likely influenced by factors that were not included in this model, Fig.3.

## DISCUSSION

In the present study, there was almost an equal distribution of medical students who used the Internet and social media sites for different purposes. Furthermore, male medical students were reported to have more social media addiction than female medical students in the current study. This finding raises intriguing questions about gender differences in digital behavior among healthcare professionals in training. This disparity can be attributed to various factors, including differences in social media usage patterns, motivation for engagement, and susceptibility to addictive online behaviours. Male students may be more prone to using social media platforms for entertainment, gaming, or networking purposes, which could potentially lead to increased time spent online and a higher risk of developing addiction. Contrary to this finding, a study in Iran reported more than 74.2% Internet use and social media addiction among female students.^12^ Social media addiction is deemed inapplicable to any gender. It relies on the physical activities, mental capabilities, and behavioral approach of students towards the use of social media apps and the internet.^13^

In the current study, 81% of participants were addicted to social media. Similarly, a study in Bangladesh found that 84% of medical students were SM addict.^14^ Furthermore, 42.2% of the students were found to use social media applications for entertainment, which diverted their concentration and affected their academic performance.^15^ The students conveyed that excessive use of cell phones during college causes them to lose concentration in class.^16^

This study highlights the severity of moderate to severe academic procrastination, stemming from poor time management, lack of motivation, fear of failure, or perfectionism. This behavior affects immediate academic performance, long-term success, mental health, and career prospects. Procrastination in academia is a widespread issue with significant consequences. Last-minute studying or rushing through assignments typically results in poor performance and increased stress, compromising work quality and limiting the depth of learning achievable through regular and timely efforts. Nevertheless, this high dependence puts the young generation at considerable risk of being affected, compromising their mental, physical, and academic activities.^17^

Similar to the study findings, the frequency of stress and its effect on students’ AP among students who live in hostels have also been reported, and medical students experience stress in Saudi Arabia.^18^ Medical students use social media as a stress mediator. However, a study in Iran reported that students living with their parents had less social media and Internet addiction, and showed better academic performance.^19^ It is essential to emphasize and evaluate students’ psychological health at home. This could help compete with challenges such as unsatisfactory academic performance and reduce excessive social media use among medical students.^20^

Procrastination refers to an increase in work delays and decrease in overall academic productivity. These findings are comparable to those of a study in Bangladesh, which reported unsatisfactory results among 35.6% of medical students. Moreover, a relevant decrease in students’ overall attendance (up to 14.1%) was observed.^14^ Regardless of dissatisfaction or absence, a positive association between SMA and AP has not been established. This could be due to significant outliers in the data. However, the findings contradicted those of the current study, as a strong relationship between Internet addiction and academic performance was observed. Unlike previous research, this study identified a strong link between internet addiction and academic performance. This divergence from earlier findings suggests that, while social media use alone may not have shown a clear impact on academic results in past studies, the broader concept of excessive Internet use demonstrates a significant relationship with academic performance in the present research.

A weak-to-moderate relationship between SMA and the number of hours disbursed was found in this study. Nevertheless, a study from Peshawar KPK reported that only 7% of medical students’ academic performance was affected when they were using social media routine.^21^ They also reported that the use of the internet for social media was up to 10 hours a day in 10% of the population. The present study estimated that several factors can account for 28.93% of the variation, and other factors could contribute. The rationale for the extreme usage of SM sites is to handle stress and anxiety.^22^ Combined with the behavioral and psychological associated with medical students, they are associated with SMA.

Consequently, educational programs that use different educational and social media applications to prevent academic failure for students are considered crucial, including application restrictions^23^, and behavioral interventions are found to be effective methods to control social media addiction.^24^ Furthermore, the parents’ role is considered an essential element in assisting their children in coping with stress and anxiety by regularly engaging with children.^25^

One of the significant causes of SMA is access to Internet facilities. However, the present research shows a weak relationship between SMA and the cost of the Internet. However, in a study in Lahore, 61% of the students reported easy access to the internet. Consequently, 70.5% of the students had access to Internet facilities at home.^26^ At the same time, the current statistics reveal that more than 90% of students in Pakistan have easy access to the Internet^27^ that could contribute to excessive social media use.

### Strengths of this study:

Data collection from multiple medical colleges and a large sample size.

### Limitations:

This study has a limitation in that the participants had a self-reported bias.

## CONCLUSION

Social media addiction and academic procrastination were high among the medical students. Parents and teachers can play an important role in educating and counseling medical students and preventing them from social media addiction to prevent them from becoming a procrastinator. The high academic scores of many medical students, regardless of their high social media addiction and academic procrastination, indicate a deep look into assessment and evaluation strategies in medical education.

### Recommendations:

Further research into gender differences in social media addiction among medical students is required. This could explore the psychological, social, and cultural factors that contribute to disparity. Understanding these patterns could inform targeted interventions and educational programs to promote healthy digital habits among future healthcare professionals and enhance their well-being and academic performance.

### Author`s Contribution:

KN: Study design, acquisition of data, manuscript writing.

KN and BJ: Concept, Literature search, critical review.

NAK: Literature search, data analysis, interpretation of data,

MJ: Concept, study design, literature search & final write-up, critical review.

All authors have read the final version and agree to be accountable for integrity of the study.
